# Effects of Brazilian Propolis on Dental Plaque and Gingiva in Patients with Oral Cleft Malformation Treated with Multibracket and Removable Appliances: A Comparative Study

**DOI:** 10.1155/2016/2038407

**Published:** 2016-09-08

**Authors:** Agnieszka Machorowska-Pieniążek, Małgorzata Skucha-Nowak, Anna Mertas, Marta Tanasiewicz, Iwona Niedzielska, Tadeusz Morawiec, Stefan Baron

**Affiliations:** ^1^Department of Orthodontics, Chair of Masticatory Dysfunction and Orthodontics, Medical University of Silesia in Katowice, Pl. Traugutta 2, 41-800 Zabrze, Poland; ^2^Department of Conservative Dentistry with Endodontics, Medical University of Silesia in Katowice, Pl. Akademicki 17, 41-902 Bytom, Poland; ^3^Department of Microbiology and Immunology, Medical University of Silesia in Katowice, Jordana 19, 41-808 Zabrze, Poland; ^4^Department and Clinic of Maxillofacial Surgery, Medical University of Silesia, Francuska 20, 40-027 Katowice, Poland; ^5^Department of Oral Surgery, Medical University of Silesia in Katowice, Pl. Akademicki 17, 41-902 Bytom, Poland; ^6^Department of Temporomandibular Disorders, Unit SMDZ, Medical University of Silesia in Katowice, Pl. Traugutta 2, 41-800 Zabrze, Poland

## Abstract

Orthodontic appliances modify the local environment of the oral cavity, increase the accumulation of dental plaque, and affect the condition of the gingiva. The aim of this study is assessment of Brazilian propolis toothpaste's effect on plaque index (PLI) and gingival index (GI) in patients with CL/CLP treated using orthodontic appliances in the 35-day study period. The study population included 96 patients of an Orthodontic Outpatient Clinic, ACSiMS in Bytom. All the patients participated in the active phase of orthodontic treatment using buccal multibracket appliances or removable appliances. During the first examination, each patient was randomly qualified to the propolis group or control group. A statistically significant decrease in GI and PLI in the entire propolis group (*P* < 0.01) was shown during repeated examination. Insignificant change in GI was in the entire control group during the repeated examination compared to the baseline. Similar result was obtained in patients treated with multibracket and removable appliances. The orthodontic appliance type did not affect the final dental plaque amount and gingival condition in patients using the propolis toothpaste. These results may be clinically useful to improve prevention and control oral infectious diseases during orthodontic treatment patients with oral cleft.

## 1. Introduction

Orthodontic appliances modify the local environment of the oral cavity, affecting the quantity, flow, composition, and biochemical properties of saliva, as well as microflora of the oral cavity [1–3]. Moreover, these appliances increase the accumulation of dental plaque and the quantity of exfoliated epithelial cells and obstruct teeth self-cleaning [4, 5]. All these factors affect the condition of the gingiva and hard tissues of the tooth and may contribute to the development of caries and periodontium diseases. In patients with cleft lip (CL) and cleft lip and palate (CLP), apart from the risk related to wearing orthodontic appliances, there are additional factors that may be responsible for poor oral health when compared to individuals with no cleft. These include oral and nasal cavity communication, contributing to mixing the microfloras of these environments and pain and fear after surgical procedures which is responsible for avoiding toothbrushing. Loss of lip elasticity in the cleft scar obstructing the access to oral cavity [6], drying mucosa, and teeth in the cleft area due to nonclosing lips and prolonged oral clearance time favouring aciduric bacteria growth [2, 7] also do not remain insignificant. Furthermore, more frequent hypoplasia and hypomineralisation of tooth enamel, presenting other locations of plaque retention, are observed in this developmental malformation compared with the general population [8].

The results of studies indicating toothpaste-containing chemical agents side effects [9, 10] have convinced the investigators to look for natural ingredients which may effectively remove dental plaque and simultaneously present a favourable effect on entire oral cavity health. One such substance includes propolis, the beneficial properties of which were already known and used in ancient times [11]. Propolis is acquired by honey bees from plant buds and tree bark splits and then enzymatically modified and used to seal the beehive entrance, line its walls, and protect against microbes [11–13]. Propolis is a thick, highly adhesive wax-resin substance composed of plant balms, essential oils, and biologically active compounds such as phenolic acid and its esters, flavones, flavonols and flavanones, aromatic aldehydes and esters, terpenes, fatty acids, *β*-steroids, mineral salts, terpenes, and vitamins (A, B1, B2, B3, and B7) [11–14]. Propolis presents strong antibacterial, antiviral, antiparasitic, antifungal, and antioxidative properties.* In vivo* and* in vitro* studies have confirmed propolis' anti-inflammatory properties and proved its strong immunomodulating effect [3, 15, 16]. Numerous scientific reports indicate propolis's beneficial effect on oral cavity health condition, yet there remain few clinical trials evaluating propolis's effect on gum hygiene and condition in patients with CL/CLP wearing different types of orthodontic appliances.

The aim of this study is the clinical assessment of ethanol extract of Brazilian propolis (EEP) toothpaste's effect on plaque index (PLI) and gingival index (GI) in patients with CL and CLP treated using buccal multibracket appliances (fixed appliances) or removable orthodontic appliances.

## 2. Materials and Methods

### 2.1. Study Population

The study material included patients of an Orthodontic Outpatient Clinic, Academic Center of Dentistry and Specialist Medicine in Bytom, Poland. All the patients participated in the active phase of orthodontic treatment using buccal multibracket appliances or removable appliances. The following inclusion criteria were used: (1) presence cleft lip and alveolus (CL) or cleft lip, alveolus and palate (CLP), (2) lack of coexisting additional developmental malformations, (3) 9–16 years of age, (4) presence of at least 10 own permanent teeth, (5) treatment using fixed buccal multibracket appliances or removable orthodontic appliance for at least 6 months, (6) no antibiotic therapy for at least 2 months prior to the study inclusion, and (7) good general health condition. Exclusions included (1) patients with bone grafting in the dental ridge of the maxilla sooner than 3 months from the study commencement and those planning these procedures in the following 2 months after qualification tests and (2) individuals with confirmed adverse reactions to bee products.

A multibracket buccal appliance (Biomim, 0.22 Roth, manufactured by Ortho Classic) was placed on at least 6 permanent teeth. A removable appliance was an acrylic palatal plate with screws for the patient to unscrew, with a labial bar, arrowhead clasps, and/or Adams clasps. Removable appliances were worn by patients for about 14 hours per day.

### 2.2. Clinical Protocol

During the first examination (baseline), every patient was provided with detailed instructions on oral cavity hygiene. Every patient was shown the method of cleaning teeth, using a set of teeth model. What is more, the patients and their parents were given spoken information regarding oral cavity hygiene and the teeth cleaning method. Each patient was recommended to clean their teeth with the Fones method, using an ordinary and an interdental toothbrush 3 times a day, and the toothpaste provided on the first visit.

During the first examination, each patient was randomly assigned, by a postgraduate students not participating in the study, to the propolis group (PG) or control group (CG). All recruits with buccal multibracket appliances and removable orthodontic appliance were qualified in a 1 : 1 ratio to one of two groups (PG, CG), so that there was a similar proportion of patients with fixed and removable appliances in both groups. PG patients were told to use toothpaste with 3% ethanol extract of Brazilian propolis, and CG patients were told to use toothpaste without an active substance, EEP (placebo). The patients were not informed which toothpaste they were given; they also could not identify it through the packaging.

#### 2.2.1. Preparation of Brazilian Green Propolis Extract

Propolis used in the study was collected from the beekeeping section of the Seiri Alimentos Naturales, Brazil. Propolis was acquired from honey bees' beehives (*Apis mellifera*) in Minas Gerais State, Southeast Brazil, from the plant* Baccharis dracunculifolia*, which is the source of resin for green propolis (propolis G12) [17]. The toothpastes with 3% of EEP and without EEP (placebo) were prepared in Nippon Zettoc Co., Ltd., Tokyo, Japan. The toothpastes used in the study were of legal origin and contained ingredients commonly used in oral cavity hygiene.

#### 2.2.2. Oral Clinical Assessment

Oral cavity hygiene and gingival condition were assessed in every patient twice, in the starting period (baseline) and after 35 days (final study). The study was conducted by a single investigator with the same illumination every time, using a mirror, a probe.

The amount of dental plaque was marked using plaque index [PLI] according to Silness and Löe [18]. Permanent superior incisors and first molars of the maxilla and the mandible were included in the study, assessing the thickness of dental plaque in the perigingival area of the tooth, using a 0–3 scale. Buccal and lingual surfaces of all examined teeth were taken into consideration. The study was performed without staining, after thorough tooth surface and its adjacent gingival margin drying using delicate air stream.

The clinical condition of the gingival margin was assessed using gingival index [GI] according to Löe and Silness [19], using the same teeth and examined surface selection criteria as with PLI. The study used a 3-step scale taking into account qualitative signs of inflammation, from 0, lack of inflammation signs, to 3, severe inflammation.

Moreover, in the case of every patient DMFT index was calculated for permanent teeth: the Decayed (D), Missing (M), and Filled (F) Teeth (T).

The study was approved by the Bioethics Committee of the Silesian Chamber of Medicine (Resolution number 6/2010). All participants were informed about the type of tests and provided written consent. The data of every patient was collected as confidential, while maintaining personal data confidentiality.

### 2.3. Statistical Analysis

Data was presented as means, SD, medians (interquartile ranges 25th, 75th percentile [IQR]). All assessed variables did not present normal distribution; therefore, nonparametric tests were used. Categorical variables were reported as proportions. Dichotomous variables were compared using chi-square tests. Comparison of scores between PG and CG was performed using Mann-Whitney *U* test. A comparison of the GI and PLI scores between the multibrackets appliance group and removable appliance group was performed using Mann-Whitney *U* test. Wilcoxon matched pairs singed-rank test was used for an intragroup comparison. The Spearman rank correlation coefficient was used to calculate the relations of gingival index and plaque index final study with initial score of gingival index, plaque index, D, M, F, T, DMFT, and type of orthodontic appliance. Statistically significant differences were considered at the level of *P* < 0.05. Statistical analysis was performed using StatSoft, Inc. (2014), STATISTICA (data analysis software system), version 12. http://www.statsoft.com/.

## 3. Results

### 3.1. Baseline

**Figure 1:**
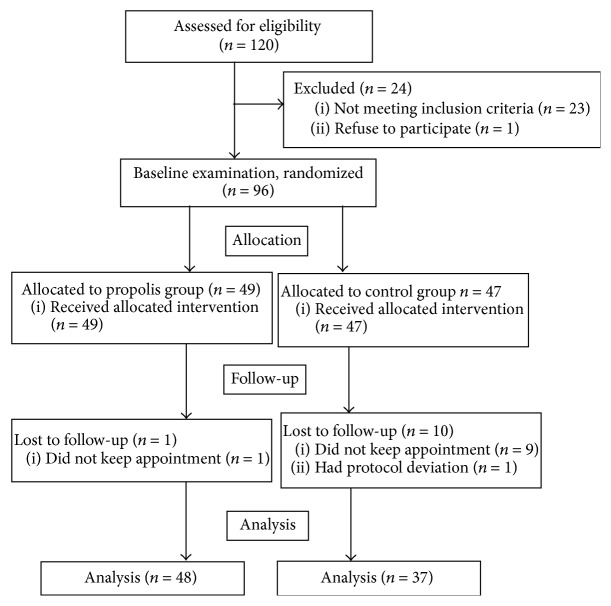
Flowchart of the progress of the phases of clinical trial.

The study involved 96 patients and was completed by 85 patients, including 33 girls and 52 boys (Figure 1). The patients from the propolis group and the control group did not differ significantly in terms of age and cleft distribution, nor orthodontic appliance type (Table 1). The median age was 12.3 in the propolis group and 11.9 in the control group. The majority of patients in both the control group and the propolis group included patients with unilateral cleft lip and palate (UCLP), 56.7% and 50.0%, respectively. Bilateral cleft lip and palate (BCLP) was present in 16.6% of patients in the propolis group and 27.0% of patients in the control group. The majority of individuals in the propolis group and in the control group were treated with buccal multibracket appliances, 64.6% and 54%, respectively. Removable appliances were present in 35.4% of the propolis group and 46% of the control group (Table 1). Mean DMFT was similar in the propolis group and the control group, being 4.45 and 4.51, respectively (Table 1). Clinical mean gingival condition expressed in GI during the first examination did not differ between both groups and was 1.5 (Table 1). Mean dental plaque amount was insignificantly higher in the propolis group compared to the control group, being 1.47 and 1.22 (*P* = 0.06), respectively (Table 1).

**Figure 2:**
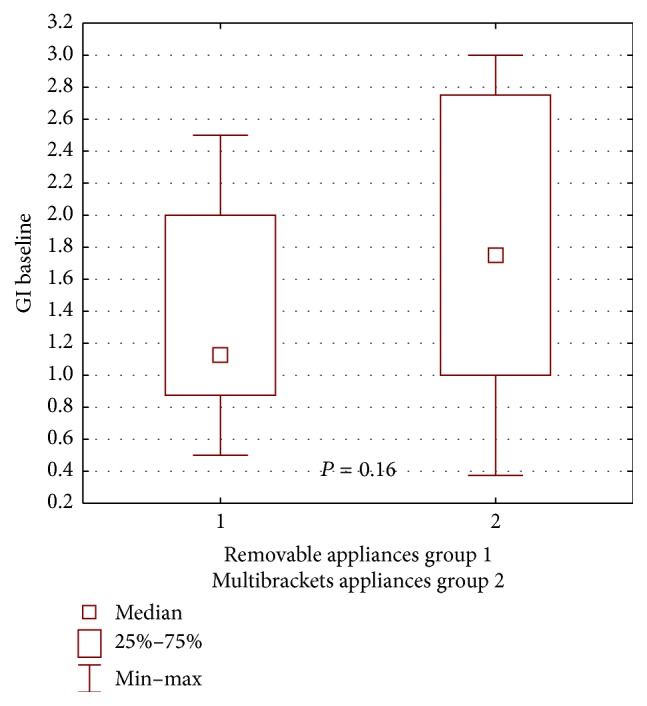
Statistical comparison of GI baseline value between removable appliances and multibrackets appliances at propolis group. Median, IQR, min, max, and significant if *P* < 0.05.

**Figure 3:**
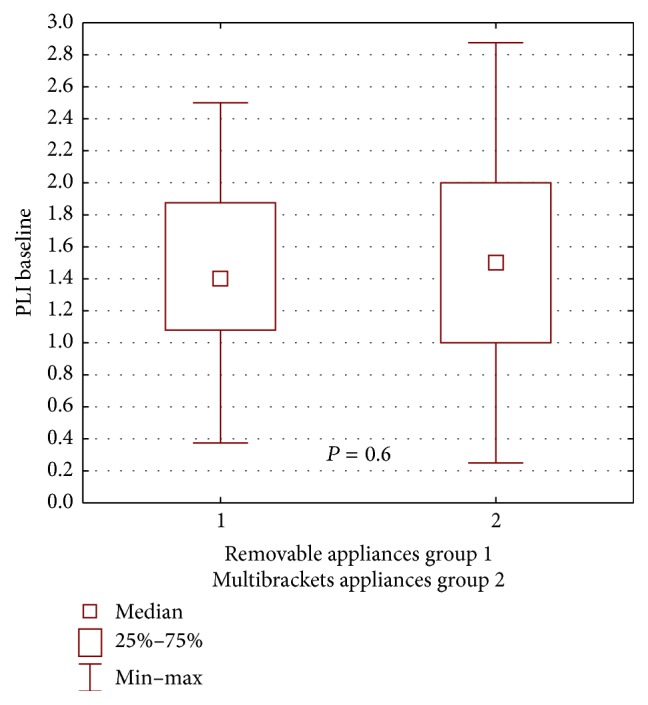
Statistical comparison of PLI baseline value between removable appliances and multibrackets appliances at propolis group. Median, IQR, min, max, and significant if *P* < 0.05.

**Figure 4:**
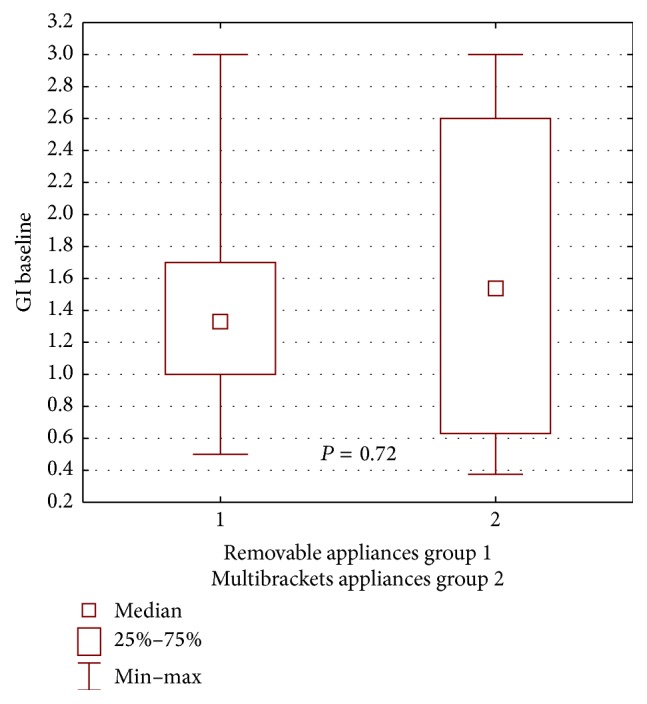
Statistical comparison of GI baseline value between removable appliances and multibrackets appliances at control group. Median, IQR, min, max, and significant if *P* < 0.05.

**Figure 5:**
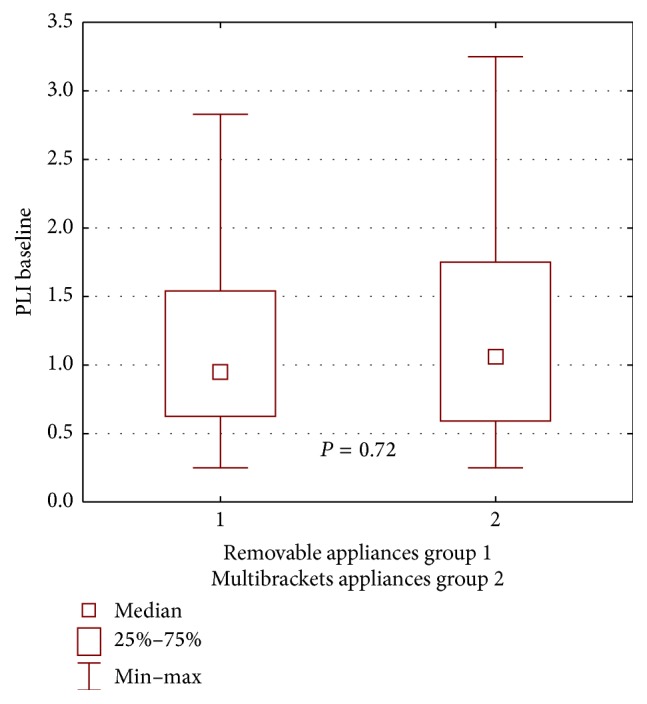
Statistical comparison of PLI baseline value between removable appliances and multibrackets appliances at control group. Median, IQR, min, max, and significant if *P* < 0.05.

During the first examination, worse clinical condition of the gingiva and the periodontium in patients wearing buccal multibracket appliances was found in both groups, compared with patients wearing removable appliances, yet the difference was not statistically significant for the propolis group: GI *P* = 0.16, (Figure 2); PLI, *P* = 0.60 (Figure 3); for the control group: GI, *P* = 0.72, (Figure 4); PLI, *P* = 0.72 (Figure 5).

### 3.2. Final Study

#### 3.2.1. Propolis Group

A statistically significant decrease in GI in the entire propolis group (*P* < 0.01) was shown during repeated examination performed after five weeks of using the propolis toothpaste (Table 2). When analysing the gingival index depending on the orthodontic appliance type, a statistically significant improvement of the gingival condition, expressed by a decrease in GI, occurred in both the buccal multibracket appliances patients group (*P* = 0.01) and the removable appliance group (*P* = 0.02) (Table 2).

During repeated examination, a statistically significant decrease in PLI and thus the amount of dental plaque were also demonstrated both in the entire propolis group (*P* < 0.01) and in patients wearing multibracket (*P* < 0.01) and removable appliances (*P* = 0.03) (Table 2).

**Figure 6:**
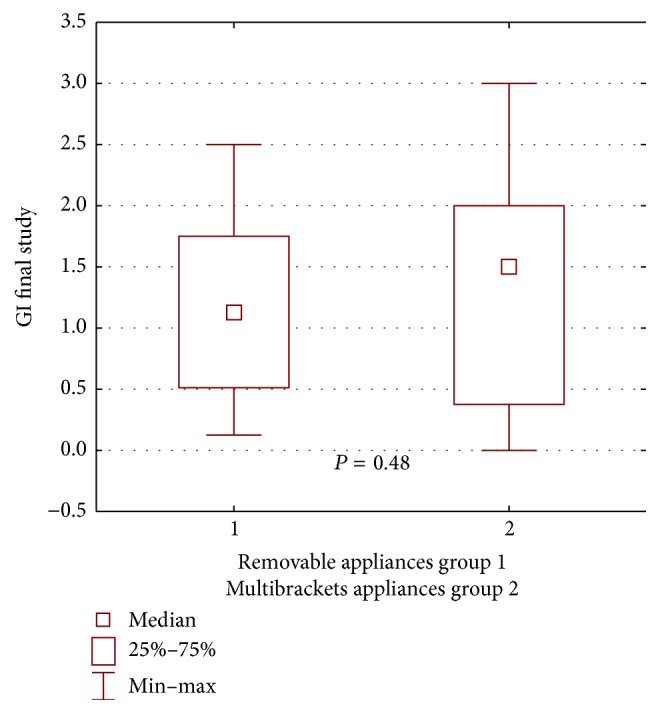
Statistical comparison of GI final value between removable appliances and multibrackets appliances at propolis group. Median, IQR, min, max, and significant if *P* < 0.05.

**Figure 7:**
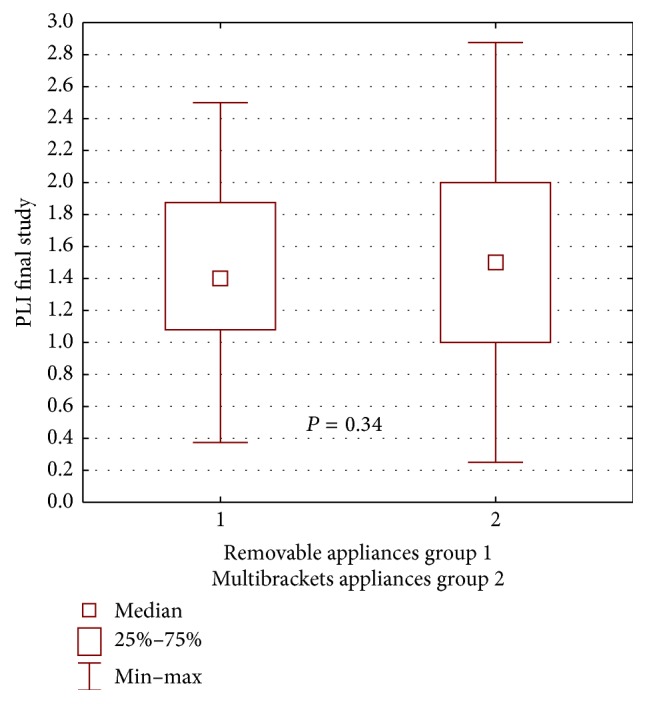
Statistical comparison of PLI final value between removable appliances and multibrackets appliances at propolis group. Median, IQR, min, max, and significant if *P* < 0.05.

A worse periodontal condition was demonstrated in patients wearing buccal multibracket appliances during the repeated examination, that is, a higher mean GI compared with patients treated with removable appliances, yet the difference was not statistically significant (*P* = 0.48) (Figure 6). A similar result was received when assessing the amount of dental plaque during repeated examination. PLI was higher in patients treated with buccal multibracket appliances, compared with patients treated with removable appliances. These differences were not statistically significant (*P* = 0.34) (Figure 7).

#### 3.2.2. Control Group

A statistically insignificant change in GI gingival index (*P* = 0.25) was found during the repeated examination in the entire control group compared to the baseline (Table 2). Similar result was obtained in patients treated with buccal multibracket appliances (*P* = 0.24) and removable (*P* = 0.64) appliances (Table 2).

A statistically significant decrease in dental plaque amount was also demonstrated between the baseline and final study in the entire control group (*P* = 0.03) and in patients treated with buccal multibracket appliances (*P* = 0.02) (Table 2). A statistically significant change in median PLI between the baseline and final study was not found in patients treated with removable appliances (*P* = 0.87) (Table 2).

**Figure 8:**
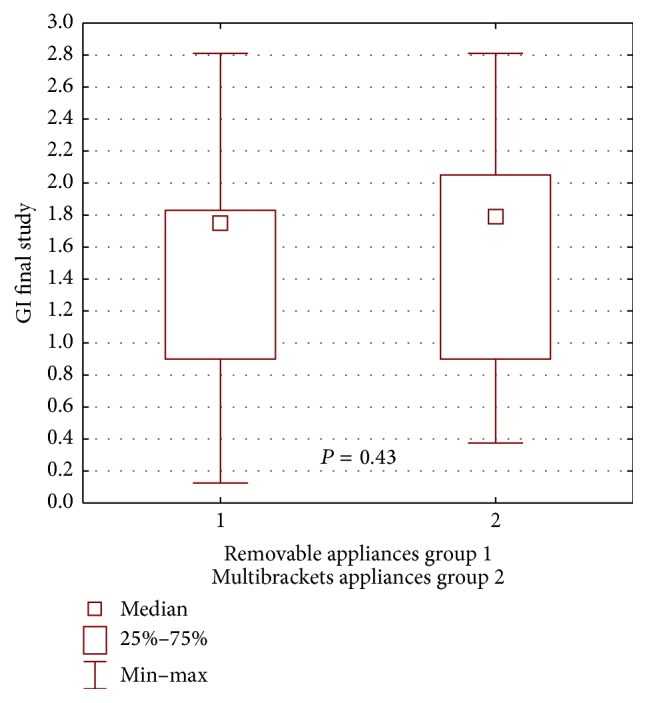
Statistical comparison of GI final value between removable appliances and multibrackets appliances at control group. Median, IQR, min, max, and significant if *P* < 0.05.

In the control group, the patients with buccal multibracket appliances had a higher median GI at the end of the study, compared with the removable appliance patients, but the difference was not statistically significant (*P* = 0.46) (Figure 8).

**Figure 9:**
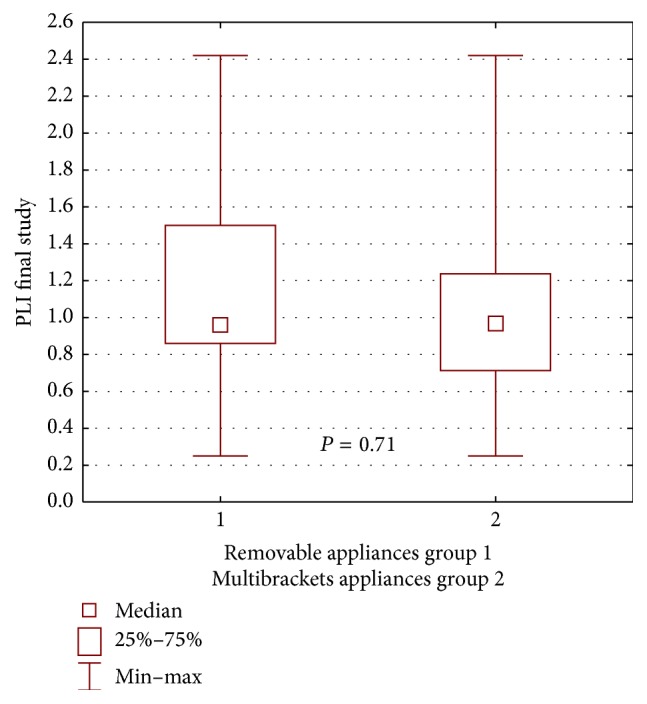
Statistical comparison of PLI final value between removable appliances and multibrackets appliances at control group. Median, IQR, min, max, and significant if *P* < 0.05.

A statistically significant difference in dental plaque amount between the buccal multibracket appliances patient group and the removable appliance group was also not found on repeated examination (*P* = 0.71) (Figure 9).

#### 3.2.3. Generally

**Figure 10:**
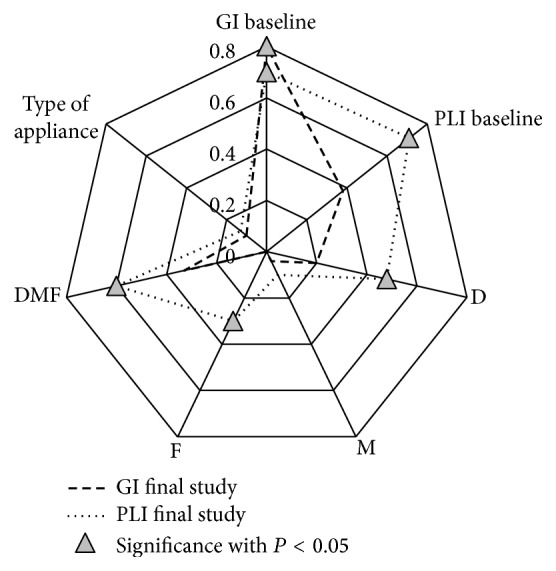
Correlation between GI baseline, PL baseline value and GI final, PLI final, Decayed, Missing, Filled Teeth, DMFT, and type of appliance in propolis group. Statistical significant correlation marked as triangle.

The analysis of final GI value correlations in the propolis group demonstrated a high statistically significant correlation with the initial GI value (Figure 10). Lack of significant correlation between final study GI and initial value of PLI, DMF and its elements, and orthodontic appliance type was also found.

**Figure 11:**
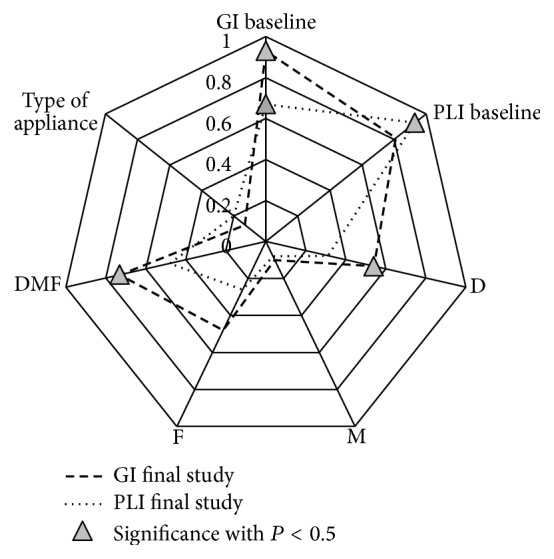
Correlation between GI baseline, PL baseline value and GI final, PLI final, Decayed, Missing, Filled Teeth, DMFT, and type of appliance in control group. Statistical significant correlation marked as triangle.

It was determined that in the control group patients there are high statistically significant correlations of final GI and PLI values with their initial values and DMFT (Figure 11).

Both toothpastes were well accepted by the patients and no adverse effect was recorded. Some patients reported taste altering and too small degree of foaming of toothpaste with propolis. The main complaint was discoloration of the toothbrush, experienced by 6 patients using toothpaste with propolis.

## 4. Discussion

Oral cavity malformation, numerous surgical procedures, and long-term orthodontic treatment are factors that may have an effect on oral cavity health in children with oral cleft. These patients are particularly at risk of developing teeth decay and inflammation of the periodontium [5, 20–22]. In the original study it was shown that the mean DMFT in children with CL/CLP is 4.45. Similar DMFT was obtained by Stec-Slonicz et al. when examining children with CL/P during active phase of orthodontic treatment [21] and Zhu et al. in patients aged 6–12 years [22]. Lower mean DMFT of 1.18 in children with cleft lip and palate was obtained by Lucas et al. [23], yet the mean age of the studied children was lower than in the original work. DMFT value applies to permanent teeth and its evaluation in younger children, with the majority of deciduous teeth, may have an effect on its lower value. Higher DMF than in the original study was demonstrated by Al-Wahadni et al. in children with CLP aged 10–15 years [24].

The gingival condition on initial examination, evaluated in the entire material through median GI, was 1.5. A higher GI value in patients with cleft lip and palate aged 10–15 years was obtained by Al-Wahadni et al. [24]. Medium and low gingivitis in patients with unilateral complete cleft were reported by Costa et al. [25]. However, it is important to emphasize that the original study included the condition of marginal gingiva in the area of superior incisors and molars. In patients with CL/CLP, the anterior segment of the maxilla is the location of cleft fissure course, scars from primary surgical procedures, and alveolar bone grafting. Furthermore, in patients with CL/CLP, the structure malformation of maxilla's alveolar processes, and vestibular fornix, fistulas to the nasal cavity may coexist with lip structure malformation. This anomaly often prevents correct lip contraction, resulting in nonphysiological drying of the maxilla alveolar processes' mucosa. The described structural and functional changes in the oral cavity in CL/CLP, as well as the methodology of the conducted research, taking into consideration only superior permanent incisors and all first molars, may have an effect on relatively high median GI obtained in this study compared with the results of other authors [20]. This view is confirmed by research conducted by Dahllof et al. who proved a statistically significant higher percentage of units exhibiting bleeding upon probing the upper anterior region, including the cleft area, in children with CL/P, compared with anterior maxillary segment in children without cleft [6].

The authors of this study examined patients with CL/CLP only, during the active phase of orthodontic treatment performed using fixed or removable appliances. Patients treated with fixed appliances presented worse oral cavity hygiene on initial examination, expressed by a higher mean PLI, and worse gingival condition expressed by a higher GI, compared with patients treated with removable appliances. The obtained results matched the research of Karkhanechi et al. who also demonstrated significantly higher PLI and GI in patients without oral cleft, treated with fixed buccal orthodontic appliances versus removable orthodontic aligners during the period between the 6th and 12th therapy month [26].

Both an improvement in gingival condition and an improvement in oral cavity hygiene were demonstrated during repeated examination in both patients using the propolis toothpaste, as well as those using the placebo. In the propolis group patients, both those treated with fixed and removable appliances, there was a statistically significant decrease in GI and PLI on final examination, compared with the initial examination. Similarly, a statistically significant PLI and GI decrease in patients using alcohol-free mouthwash containing 5.0% Brazilian green propolis over 45 and 90 days was reported by Pereira et al. [27]. Tanasiewicz et al. presented a beneficial propolis effect on oral cavity condition in patients with gingivitis and those with periodontitis [28], whereas Morawiec et al. presented a similar beneficial propolis effect in patients with implants [29].

The results of this study showed a lack of correlation between gingival condition in the final examination and initial oral cavity hygiene and DMFT intensity in propolis group. Therefore, the original study found that propolis masks the effect of initial plaque accumulation and DMFT on gingival condition in the final examination. This effect may indicate a beneficial propolis effect on gingival condition both in children with satisfactory and poor oral cavity hygiene and in those with high and low tooth decay intensity.

Brazilian propolis was used in the study as the active toothpaste component. The compounds present in Brazilian propolis include cinnamic acid and derivatives, coumaric acid, prenylated compounds of chroman and chromen, substituted phenolic esters, flavonoids (flavones, flavanones, and flavonols), benzoic acid, benzopirans, dihydrobenzofurans, and benzofurans [30–32]. Among the cinnamic acid derivatives of Brazilian propolis, artepillin C is the best known [30–33]. According to the reviewed specialist references, artepillin C is one of the components responsible for the antibacterial, antineoplastic, anti-inflammatory, and immunomodulating properties of propolis [34].

The research performed on an animal model showed that the anti-inflammatory action of artepillin C consists of, for example, inhibiting tissue swelling and decreasing total neutrophil count, as well as decreasing prostaglandin E2 level in peritoneal fluid [34]. The mechanism of artepillin C anti-inflammatory action was also confirmed through* in vitro *tests in which the concentration of nitrite in the supernatant in macrophage cell culture decreased as an effect of artepillin C [34]. The permeability of blood vessel capillaries increases during the inflammatory process in the gingiva which promotes plasma protein penetration and causes tissue swelling. Apart from the colour change, it is the second symptom grading the gingivae inflammatory process intensity, assessed in this study using GI index. A statistically significant decrease in the mean value of this index confirms propolis's effect on periodontal inflammation suppression and its effectiveness as an active toothpaste component in patients with CL/CLP.

Detailed studies of propolis's properties have shown that ethanol extract of propolis (EEP) is able to regulate gene expression of bacteria and can cause changes in the bacterial protease activity [35]. The statistically significant PLI and GI decrease during the final study in patients using propolis toothpaste, obtained in the original research, may be related to modifying the maturation and differentiation of bacterial biofilms. Propolis's effect of modifying the dental plaque composition may explain the lack of correlation between final gingivae condition and initial residue amount. This is why it is also effective in patients with high initial plaque accumulation. Propolis's antibacterial action in patients with CL/CLP was also confirmed in the study author's works regarding microflora of the oral cavity [36]. In the control group, the gingival condition in the final study worsens with the increase of initial dental plaque amount; therefore, GI increases.

The ingredients of commonly used toothpastes mostly include chemical compounds such as chlorhexidine [37], triclosan [38], fluorine compounds [39–41], peroxides and hydrogen peroxide [42], cetylpyridinium chloride [38], sodium lauryl sulphate [43, 44], and the salts of metals [45]. Some of these substances may penetrate into the organism through the mucosa, and also as a result of swallowing [46], and disrupt the metabolism of the oral cavity's epithelium [9, 10]. Propolis as a natural substance has very few side effects, which can be minimized by removing the constituents responsible for allergy. However, the incidence of beekeeper's allergic contact dermatitis due to topical application of propolis and adverse reactions due to propolis ingestion are described [47].

Propolis is relatively low-cost, nontoxic and has mucoprotective properties for oral and gastric mucosa [48]. There are no reports dealing with bacterial resistance to propolis [49]; moreover, synergism between propolis and antibacterial agents has been observed [50]. These findings support view that propolis is a promising therapeutic agent in prevention of oral diseases caused by microorganisms.

According to the results of the study, it should be stressed that toothpaste containing propolis is suitable for improvement gingival condition and elimination of the dental plaque and might be used as a potential anti-inflammatory preparation for infectious oral diseases. However, it needs to be emphasized that further step should be given to remove discoloration properties and to improve organoleptic features of studied toothpaste, including more accepted colour, smell, and taste.

## 5. Conclusions

The conducted study showed that propolis, as an active toothpaste component, may be an effective agent used in oral cavity hygiene in patients with CL/CLP treated with both fixed and removable appliances. The orthodontic appliance type did not affect the final dental plaque amount and gingival condition in patients using the propolis toothpaste. Propolis's action was visible through an improvement in gingivae condition, as well as oral cavity hygiene. The final study decrease in gingival inflammation symptoms in patients using the propolis toothpaste was not related to the initial amount of dental plaque and dental caries intensity. In fact it was quite the opposite: in patients using the placebo toothpaste, the final gingival condition was highly correlated with the initial amount of dental plaque. Instruction on oral cavity hygiene, used on its own, is an effective method of improving oral cavity hygiene in patients with fixed appliances, yet it had no significant effect on gingival condition in the 35-day study period.

## Figures and Tables

**Table 1:** Characteristic of propolis group and control group; intergroup differences.

	Propolis group			Control group			Intergroup difference *P*
	Female	Male	Total	Female	Male	Total	
	*n* = 18	*n* = 30	*n* = 48	*n* = 15	*n* = 22	*n* = 37	
Age in years							
Median	12.6	12.3	12.3	12,43	11,62	11,90	
25th–75th percentile (IQR)	11.2–13.6 (2.4)	10.9–14.7 (3.8)	11.14–14.1 (2.9)	10.7–13.8 (3.1)	9.8–12.8 (3.0)	10.3–13.1 (2.8)	*0.375* ^a^

UCLP *n* (%)	9 (18.7%)	15 (31.3%)	24 (50%)	8 (21.6%)	13 (35.1%)	21 (56.7%)	*0.536* ^b^
BCLP *n* (%)	3 (6.2%)	5 (10.4%)	8 (16.6%)	3 (8.1%)	7 (18.9%)	10 (27%)	*0.955* ^b^
UCL *n* (%)	6 (12.5%)	10 (20.9%)	16 (33.4%)	4 (10.8%)	2 (5.4%)	6 (16.2%)	*0.246* ^b^

Multibracket appliances *n* (%)	10 (20.9%)	21 (43.7%)	31 (64.6%)	8 (21,6%)	12 (32.4%)	20 (54.0%)	*0.074* ^b^
Removable appliances *n* (%)	8 (16.7%)	9 (18.7%)	17 (35.4%)	7 (19.0%)	10 (27.0%)	17 (46.0%)	*0.325* ^b^

DMFT							
Mean (SD)			4.45 (3.55)			4.51 (4.48)	
Median (IQR)			5.0 (6.0)			5.0 (5.0)	*0.22* ^a^

GI							
Mean (SD)			1.58 (0.85)			1.52 (0.87)	
Median (IQR)			1.5 (1.2)			1.3 (1.3)	*0.67* ^a^

PLI							
Mean (SD)			1.47 (0.62)			1.22 (0.83)	
Median (IQR)			1.5 (1.0)			0.96 (1.0)	*0.06* ^a^

IQR, interquartile range; ^a^ *U* Mann-Whitney test; ^b^chi-square test, ^*∗*^significance if *P* < 0.05.

**Table 2:** Plaque index (PLI) and gingival index (GI) scores of patients propolis group and control group treated with multibrackets or removable appliances; intragroup differences between baseline and final study.

		Propolis group			Control group		
		Median (IQR)		Intragroup difference	Median (IQR)		Intragroup difference
		Baseline	Final study	*P* ^*∗*^	Baseline	Final study	*P* ^*∗*^
GI	Multibracket appliances	1.75 (1.75)	1.5 (1.62)	0.01^*∗*^	1.54 (1.97)	1.79 (1.15)	0.24
	Removable appliances	1.12 (1.25)	1.12 (1.23)	0.02^*∗*^	1.33 (0.70)	1.75 (0.93)	0.64
	Total	1.50 (1.25)	1.25 (1.37)	<0.01^*∗*^	1.33 (1.37)	1.75 (1.02)	0.25

PLI	Multibracket appliances	1.50 (1.0)	1.50 (1.04)	<0.01^*∗*^	1.06 (1.15)	0.96 (0.52)	0.02^*∗*^
	Removable appliances	1.40 (0.79)	1.08 (0.79)	0.03^*∗*^	0.95 (0.91)	0.96 (0.64)	0.87
	Total	1.50 (1.0)	1.25 (1.0)	<0.01^*∗*^	0.96 (1.0)	0.96 (0.42)	0.03^*∗*^

IQR, interquartile range; ^*∗*^significance if *P* < 0.05.
